# Mucocele of the Appendix: A Presentation

**DOI:** 10.1055/s-0042-1743516

**Published:** 2022-09-19

**Authors:** Mahendra Pratap Singh, Tanweerul Huda

**Affiliations:** 1 Department of General Surgery, All India Institute of Medical Sciences, Bhopal, India; 2 Department of General Surgery, LN Medical College, Bhopal, India

**Keywords:** mucocele, appendix, epithelial tumors

## Abstract

The mucocele of the appendix can be described as an obstructive dilatation of the appendix by an intraluminal accumulation of mucus. A 60-year-old diabetic male patient presented with chief complains of pain in right lower abdomen for the past 2 months which was dull in nature, not associated with fever, vomiting, diarrhea, constipation, or any urinary complains. Contrast-enhanced computed tomography (CECT) of the abdomen revealed appendiceal lumen distended, filled with fluid collection. There was abrupt narrowing seen at its junction with cecum. Features were suggestive of appendicular mucocele. The patient was taken up for exploratory laparotomy, and a distended turgid appendix, around 4 cm in diameter with dilated cecum, was found. Ileocecal resection was done followed by ileo-ascending colon side-to-side anastomosis using staplers. The histopathological examination report revealed an R0 resection. The patient was followed up for 3 years postoperatively with CECT of the abdomen and a colonoscopy yearly. There was no evidence of any recurrence in the follow-up.

The mucocele of the appendix can be described as an obstructive dilatation of the appendix by an intraluminal accumulation of mucus. 1 2 3 It can be caused by these four etiologies: retention cysts, mucosal hyperplasia, cystadenomas, and cystadenocarcinomas. Clinically, it has a nonspecific presentation. It is most commonly found incidentally during appendicectomy. The principles of surgery for appendiceal mucocele include resection of the appendix, wide resection of mesoappendix including all appendiceal lymph nodes, collection and cytologic examination of the mucus, and inspection of the base of the appendix. 1 2 3 In case of positive margin at the base or positive periappendiceal lymph nodes, right hemicolectomy or ileocecectomy is warranted. 3 Pseudomyxoma peritonei (PMP) is a dreaded complication of rupture of mucocele appendix intraperitoneally. It is characterized by peritoneal tumor deposits, mucinous ascites, omental caking, and ovarian involvement in females. The risk for developing PMP after appendicectomy having epithelial tumor is around 9%. 1

## Case Presentation

**Fig. 1:**
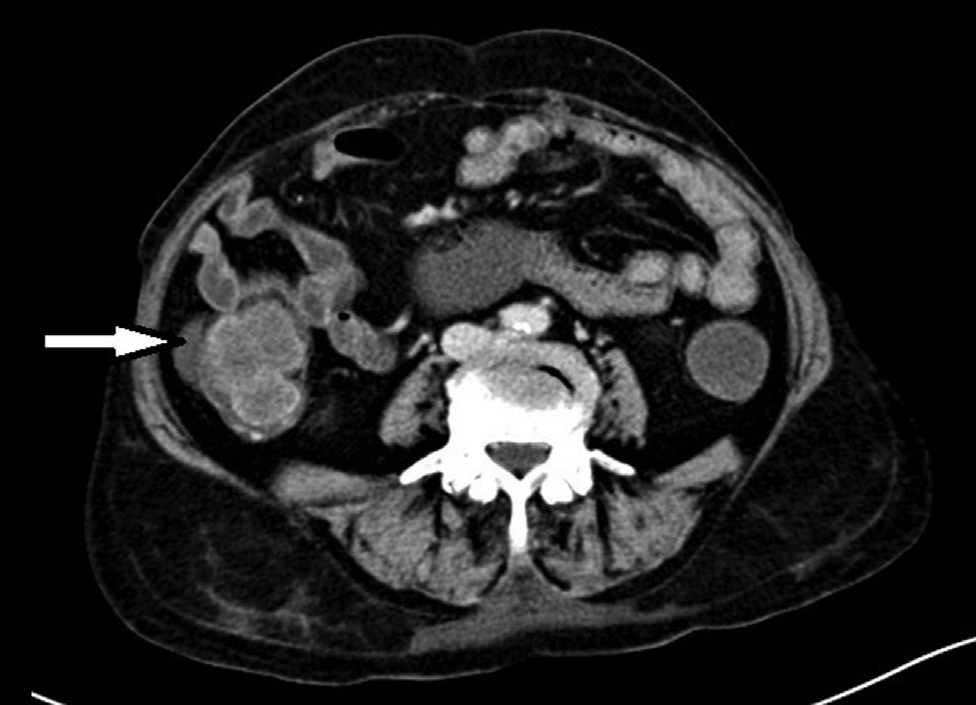
Contrast-enhanced computed tomography of the abdomen showing dilated appendix.

**Fig. 2:**
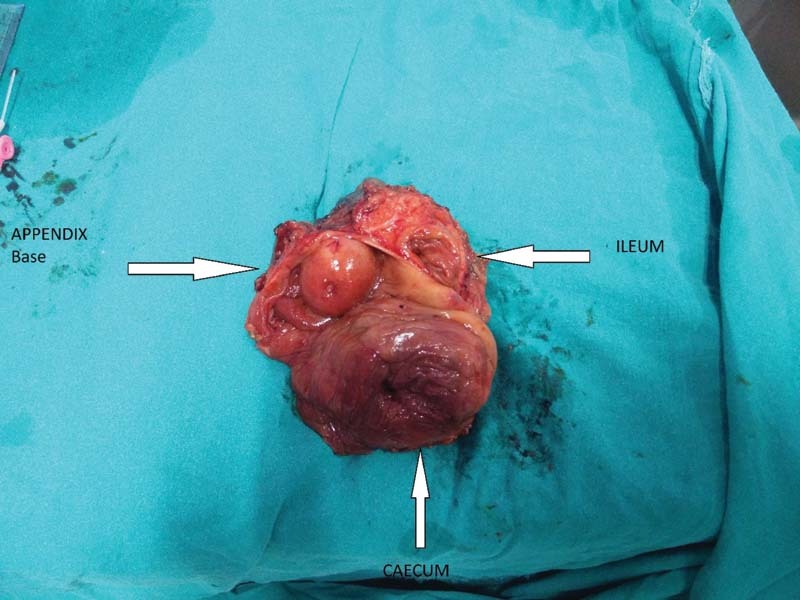
Resected specimen showing appendiceal opening with cecal and ileal opening.

**Fig. 3:**
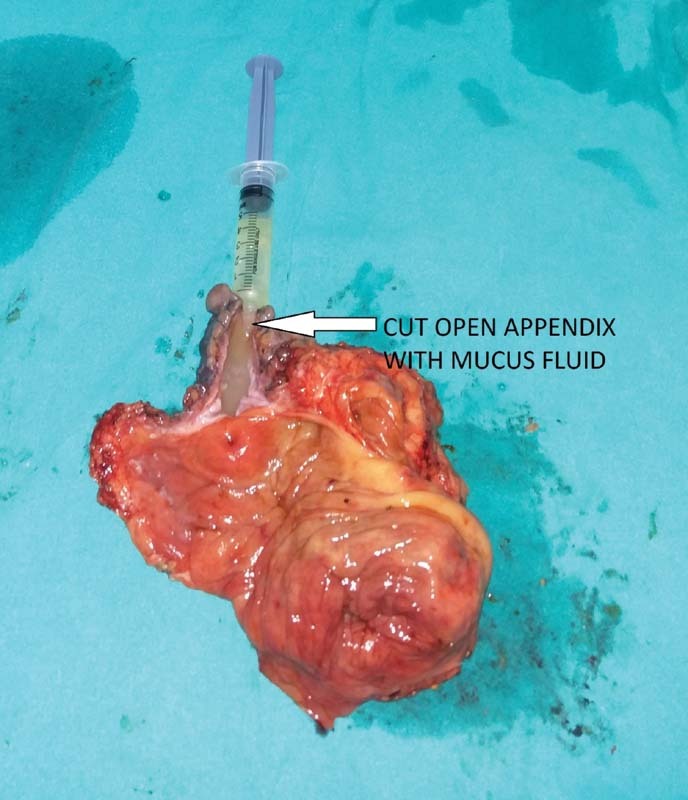
Resected specimen showing cut open appendix with mucous fluid content.

A 60-year-old diabetic male patient presented with chief complains of pain in right lower abdomen for the past 2 months which was dull in nature, not associated with fever, vomiting, diarrhea, constipation, or any urinary complains. There was no associated history of loss of appetite or weight loss and no history of hematemesis or melena. The patient was taking treatment on and off but was not relieved. Per abdomen, examination revealed a lump in the right iliac fossa region. All routine laboratory investigations were unremarkable. Upper gastrointestinal (GI) endoscopy was unremarkable. Colonoscopy showed edematous appendiceal lumen indenting into the cecum. No other abnormality was detected. Ultrasonography of the abdomen revealed a dilated (15 mm), fluid-filled, blind-ending, partially compressible, nonperistaltic bowel loop in the right iliac fossa. The features were suggestive of appendiceal mucocele. Contrast-enhanced computed tomography (CECT) of the abdomen revealed appendiceal lumen distended, filled with fluid collection (iodine-related Hounsfield unit [IHU] value 9), measuring 2.8 cm in diameter at its base with the appendiceal wall thickness of 3.5 mm ( Fig. 1 ). There is abrupt narrowing seen at its junction with cecum. No obvious enhancing nodular lesion/appendicolith was noted. No significant periappendiceal fat stranding or fluid collection or adjacent enlarged lymph nodes were seen. Features were suggestive of appendicular mucocele. The patient was taken up for exploratory laparotomy and a distended turgid appendix, around 4 cm in diameter with dilated cecum was found. Ileocecal resection was done followed by ileo-ascending colon side-to-side anastomosis using staplers ( Figs. 2 and 3 ). The postoperative stay was uneventful. Mucocele fluid cytology report showed abundant macrophages along with inflammatory cells comprising lymphomononuclear cells and polymorphonuclear cells in the background showing thick mucoid proteinaceous material. No atypical cells were seen. Histopathological examination report revealed grossly enlarged and dilated appendix. The base of the appendix showed narrow but patent lumen with mucosa bulging into the cecal lumen. Focally lumen of the appendix showed thick gelatinous mucinous substance. Section from appendix showed mucosa lined by flattened columnar mucinous epithelium without significant dysplasia, thinned out wall with loss of lymphoid tissue and submucosa. Underlying stroma showed mild to moderate chronic inflammatory infiltrate. Sections taken from the appendix near the opening at ileocecal junction showed dissection of acellular mucin in the wall up to muscularis propria. Features were suggestive of low-grade appendiceal mucinous neoplasm (LAMN).

The histopathological examination report revealed an R0 resection. The patient was followed up for 3 years postoperatively with CECT of the abdomen and a colonoscopy yearly. There was no evidence of any recurrence in the follow-up. No specific GI symptoms were seen.

## Discussion

Appendiceal neoplasms are found in around 1% of appendicectomy specimens. Most of them are found incidentally. Tumors of the appendix can be classified as carcinoid or epithelial. Epithelial tumors of the appendix account for around 75% of the cases. Carr et al classified the epithelial tumors broadly as mucinous or nonmucinous (intestinal type). Their classification included adenomas (tubular, tubulovillous, villous), serrated polyp, nonmucinous adenocarcinoma, mucinous neoplasm (LAMN, high-grade appendiceal mucinous neoplasm, and mucinous adenocarcinoma), adenocarcinoma with signet ring cells and signet ring carcinoma. Based on the degree of cytologic atypia and architectural features, it was classified as infiltrative or pushing invasion. 1 2

Goblet cell carcinoid, a rare tumor of the appendix, has now been recently reclassified as a goblet cell tumor. It can be of mucinous or nonmucinous type. Goblet cell tumor has both gland forming and neuroendocrine features. 1 4 Carcinoid tumors arise in argentaffin tissue (kulchitsky cells of the crypts of lieberkuhn). They are most commonly seen in the appendix. It can occur in any part of the appendix, but most commonly found in the distal one-third. Grossly on the cut surface, it is seen as a yellow mass between the intact mucosa and peritoneum. Carcinoid tumors of the appendix rarely metastasize. 1 4

Treatment of mucocele appendix is appendicectomy primarily. Frozen section of the base of appendix intraoperatively can help in distinguishing the mucocele from other mucinous neoplasms. In case of simple mucocele and less than 2 cm in diameter, an appendicectomy with removal of all fat and lymph nodes in mesoappendix is warranted. However, in the case of positive margin at the base, dilated appendicular base more than 2 cm or positive periappendiceal lymph nodes, right hemicolectomy, or ileocecectomy is warranted. 3 5 There are various reports of association of mucocele of an appendix with colorectal tumors and ovarian mucinous tumors. So, colonoscopy needs to be done in all patients preoperatively as well as postoperatively in follow-up. 4 6 7

Patients having low-grade epithelial neoplasms without any evidence of mucin or epithelial cells beyond the appendix have a very low risk of PMP development. A colonoscopy should be done to exclude any associated colonic epithelial lesions and patients kept in surveillance postoperatively for at least 5 years. Surveillance may include clinical review, annual abdominopelvic CT scan, and appendix-related tumor markers (CEA, CA 199, CA 125). 1

Patients having a high-grade tumor, invasive adenocarcinoma or goblet cell tumor, and/or those with an epithelial cell containing mucin outside the appendix have a higher risk of nodal involvement and subsequent development of PMP. These patients should be treated as patients with established PMP and considered for right hemicolectomy with prophylactic regional (right parietal) peritonectomy, omentectomy, and intraperitoneal chemotherapy. They should also be considered for bilateral salpingoophorectomy where feasible. 1

Treatment of PMP is cytoreductive therapy combined with hyperthermic intraperitoneal chemotherapy. Cytoreductive therapy is achieved by multiple peritonectomy procedures along with multiple visceral resections. 1 8 9 10

## Conclusion

Mucoceles of the appendix are most commonly found incidentally during an appendicectomy. Frozen section of the base of appendix intraoperatively provides vital clues to distinguish it from other mucinous neoplasms. Ileocecectomy can be done as a single-stage procedure when anticipating the appendiceal base involvement during exploration or in case of high suspicion in CECT of the abdomen for mucocele appendix. Laparoscopic appendicectomy can also be performed for mucocele of the appendix, but the risk of rupture and further complications of a PMP must be kept in mind.
